# Attitudes of prehospital providers on transport decision-making in the management of patients with a suicide attempt refusing care: A survey based on the *Mental Health Care Act* of 2002

**DOI:** 10.4102/sajpsychiatry.v24i0.1156

**Published:** 2018-10-30

**Authors:** Katya Evans, Heike Geduld, Willem Stassen

**Affiliations:** 1 Department of Emergency Medicine, University of Cape Town, South Africa; 2 Department of Health, Mitchells Plain District Hospital, South Africa

## Abstract

**Background:**

Given the frequency of suicidal patients making attempts prior to a completed suicide, emergency access to mental health care services could lead to significant reduction in morbidity and mortality for these patients.

**Aim:**

To describe the attitudes of prehospital providers and describe transport decision-making around the management of patients with a suicide attempt.

**Setting:**

Cape Town Metropole.

**Methods:**

A cross-sectional, vignette-based survey was used to collect data related to training and knowledge of the *Mental Health Care Act*, prehospital transport decision-making and patient management.

**Results:**

Patients with less dramatic suicidal history were more likely to be discharged on scene. Few respondents reported the use of formal suicide evaluation tools to aid their decision. Respondents displayed negative attitudes towards suicidal patients. Some respondents reported returning to find a suicidal patient dead, while others reported patient attempts at suicide when in their care. Eighty per cent of respondents had no training in the management of suicidal patients, while only 7.0% had specific training in the *Mental Health Care Act*.

**Conclusion:**

A critical lack in the knowledge, training and implementation of the *Mental Health Care Act* exists amongst prehospital providers within the Western Cape. A further concern is the negative feelings towards suicidal patients and the lack of commitment to transporting patients to definitive care. It is essential to urgently develop training programmes to ensure that prehospital providers are better equipped to deal with suicidal patients.

## Introduction

‘People who are suicidal need further management – to be left alone is like saying nobody cares.’… ’[*I*] wish further training could be done as most patients end up DOA [*dead on arrival*] at a later stage.’ (Participant 38, female, provincial sector)

Globally, suicide poses an increasingly grim public health concern and is the 13th leading cause of mortality worldwide.^1^ The World Health Organization (WHO) estimates 1 million deaths occur annually as a result of suicide.^2^ In South Africa, national suicide figures in the year 2016 give age-standardised rates of 18.7/100 000 for men and 4.7/100 000 for women.^3^

In society, there is a deep-seated belief that people who threaten suicide are not likely to follow through with their threat. However, the data assert that this is incorrect.^4^ Repeated suicide attempts often occur when a patient’s initial threat or attempt does not get the desired effect (i.e. the cry for help fails).^4^ About 10% of those who threaten or attempt suicide eventually do kill themselves,^5^ while 80% of those who commit suicide have previously verbally stated their intention to do so.^6^

Refusal of treatment in the emergency setting by patients who have attempted or threatened to commit suicide is a common problem for health care workers. Negative preconceptions and the strong social stigma surrounding mental-health-related hospitalisations may lead to patients refusing treatment.^7^ Patients may perceive a punitive or threatening environment and expect difficulties in health worker-patient relationships.^8^ Patients discharged against medical advice (DAMA) showed reduced treatment benefit; worse psychiatric, medical, psycho-social and socioeconomic functioning; had decreased access to outpatient services; overused emergency services; and were readmitted sooner.^8^ Considering the risks of multiple suicide attempts, the detection and follow-up of treatable conditions is key to successful long-term management of patients at risk.^5^

Kuo et al.’s study on 11 040 acutely ill psychiatric inpatients in Taiwan found an increase in incidence of successful suicide in psychiatric patients who leave ‘DAMA’ compared to those who were discharged by their psychiatrists.^7^ Patients who were DAMA might have unresolved, unaddressed detrimental psychiatric or psychosocial difficulties and were more likely to complete suicide.^7^ Patients presenting to health care services with a suicide attempt who receive active suicide prevention contact and follow-up may reduce the risk of repeated suicide attempts in the next year.^9^

First responders and prehospital personnel have been identified by the WHO as key role-players in the prevention of community suicide.^2^ Prehospital providers are often the first and only health care providers on scene after a suicide attempt; thus, their role in the patient’s access to mental health care services is critical. Of concern is the lack of formal training in the *Mental Health Care Act* (*MHCA*) and direction by policy-makers for these practitioners. The urgency of managing these patients within the community adds additional cognitive and emotional stress for prehospital providers.^5^ Prehospital providers engage in the management of the suicidal patients in two ways: most commonly in the assessment at the scene of the incident and transport to hospital, as well as the interfacility transfer of patients to specialised psychiatric services.^10^

South Africa has a three-tiered ‘level of care’ emergency medical services (EMS) model, basic life support (BLS), intermediate life support (ILS) and advanced life support (ALS). The South African EMS is currently going through a period of transition. Until now, the pathway to become a prehospital provider was either through vocational short-course training, or through formalised tertiary education. After 6 weeks of training, an individual could register with the Health Professions Council of South Africa (HPCSA) as a Basic Ambulance Assistant (BLS). After approximately 6 months of work experience, a BLS provider could complete a 6–8-month course, registering as an Ambulance Emergency Assistant (ILS). Finally, after another 6 months of work experience, the ILS provider could complete a 9–12-month course and register as a Critical Care Assistant (ALS). This means of qualification is now being phased out, as promulgated by the Minister of Health of South Africa. Going forward, formal academic training at a higher education institute is required to register as a prehospital provider. The courses are between 1 and 4 years in duration. The overarching term ‘prehospital provider’ has been selected to describe any EMS practitioner in this article, regardless of qualification.

In a resource-limited health system, first responders frequently become the gatekeepers for access to mental health care services and, as a result, they need to have a clear understanding of both local legislation for involuntary assessment and treatment criteria.^11^ These individuals can frequently become discouraged from transporting patients to health care facilities because of long waiting times and bed shortages, and these frustrations undermine the importance of helping the patient access mental health care and substance abuse treatment.^2^ Pre-arranged agreements between prehospital services, hospitals, community mental health care services and addiction agencies can help the first responder to streamline the referral process.^2^

Patients who have attempted to commit suicide may have limited insight into their illness and a restricted ability to cooperate with treatment.^12^ This may require measures that, in line with local legislation, restrict their personal freedom and allow transportation to hospital against their will.^5^ However, the health protocols on the evaluation of suicidal patients rarely apply to prehospital providers, who are generally the first at the scene.^5^

Actively suicidal, aggressive or agitated patients can pose a risk to staff safety on the scene of an incident. Pre-hospital providers have limited training around verbal de-escalation, and should verbal de-escalation techniques fail and patients pose a danger to themselves; prehospital providers may require the assistance of ALS paramedics able to prescribe and administer sedative drugs, physical restraints and/or police intervention may be required to transport patients to a facility for a formal risk assessment.^5^ Safety is also of concern during ambulance transport. There have been cases reported of injury or completed suicide during hospital transfer because of patients jumping from moving ambulances.^11^

In addition to the explicit guidance on the process for application for involuntary admission of psychiatric patients, the South African *MHCA* of 2002 provides criteria to which the patient should be assessed in order to initiate such an application. In short, the patient should be incapable of making an informed decision regarding their own care because of suspected mental illness, should pose a threat of harm to themselves or others, and care is needed to protect the financial interests or reputation of the individual. Such applications should be made in writing.^13^ For prehospital providers, this is not achievable at the scene and therefore requires the rapid assessment and transport of these patients to health facilities for evaluation and possible emergency admission on an involuntary basis. The *MHCA* describes such involuntary transport to be a function of the South African Police Services and does not address the role of EMS. In reality, however, EMS are often the first to arrive at the scene of a suicide attempt and have to manage these patients without police assistance. Determining the training received on and the interpretation and application of the *MHCA* by prehospital providers is therefore essential towards ensuring adequate care for suicidal patients.

### Aim

The aim of this study was to describe the attitudes (perceptions and experiences) of prehospital health care providers regarding the care and management of patients with a suicide attempt in the Cape Town Metropole.

### Objectives

The following were the objectives of this study:

- to understand the perceptions of prehospital providers regarding transport decisions for suicidal patients
- to describe personal experiences of the management of patients with suicide attempts
- to determine the training that prehospital providers have received on the *MHCA* in dealing with mental health care users.

## Research methods and design

The study was a cross-sectional design using a survey with open- and closed-ended questions. This survey was generated through a review of the literature and validated for content by an expert group of emergency medicine, prehospital medicine and psychiatry practitioners.

The survey included demographics, training in and knowledge of the *MHCA* in relation to prehospital transport decision-making. There were also five vignettes in which respondents were expected to describe their transport and management decisions in various patients who refused transportation. The vignettes ranged in severity of suicide attempt based on traditional risk factors (patient age, gender, major depression, feelings of hopelessness, substance abuse and previous suicide attempts).^14^ Opinions regarding suicidal patients were explored, as well as accounts of challenges they have experienced transporting suicidal patients.

A pilot was carried out with a small cohort of prehospital providers to test usability and feasibility. A convenience sample of 100 prehospital providers of all levels from the provincial and private sector was sought via cluster randomisation of Cape Town ambulance stations. Inclusion criteria were registration with the HPCSA and full-time clinical operational employment.

Data were subjected to descriptive analysis with the aid of NVivo^®^ software (QSR International; Victoria, Australia) as well as hand coded independently by the three researchers. Demographic data and multiple-choice questions are presented as total numbers, means, medians and standard deviations. Associations between demographic data, knowledge-based answers and transport decisions were investigated by chi-square analysis.

## Ethical consideration

Ethics approval was obtained from the University of Cape Town’s Human Research Ethics Committee (HREC 533/2014) and local permission to conduct the study was obtained from the three ambulance services’ relevant research committees.

## Results

One-hundred and thirty paper surveys were distributed and 100 were returned, yielding a response rate of 77.0%. Two responses were excluded for data quality, with 98 responses eligible for analysis.

The majority of respondents were men (65.0%), between 20 and 30 years (44.0%), with 1–10 years of experience (74.0%). There was an equal distribution of private and public service respondents (Table 1).

**TABLE 1:** Sample description (*n* = 98).

Variable	*n*	%
**Gender**		
Male	64	65
Female	34	35
**Age (years)**		
20-30	43	44
30-40	35	36
40-50	11	11
50-60	2	2
Blank	7	7
**Years of experience**		
< 1	6	6
1-5	36	37
5-10	36	37
➢ 10	20	20
**Work sector**		
Government	49	50
Private	48	49
Unassigned	1	1
**Qualification**		
BAA	24	24
AEA	47	48
ECT	7	7
CCA	5	5
NDip	12	12
BTech/BEMC	3	3

BAA, Basic ambulance assistant (basic life support); AEA, ambulance emergency assistant (intermediate life support); ECT, emergency care technician (intermediate life support); CCA, critical care assistant (advanced life support); NDIP, National Diploma in Emergency Medical Care (advanced life support); BTECH / BEMC, Bachelor’s Degree Paramedic (advanced life support).

### Feelings around transport decisions

Common themes were identified in the answers to case vignettes (Box 1). While the risk to self-harm was more explicit, the practitioners reported a higher need for police involvement and the use of physical and chemical restraint. In younger patients, family involvement was more likely. In less than two-thirds of all vignettes did participants take steps to convince patients to be transported to hospital. Some respondents reported utilising some form of informal risk assessment methods, with no practitioners reporting the use of validated risk assessment tools. Another common theme identified was the described need for a more senior role-player to aid with transport decision-making. This ranged from the control room supervisor, officer or shift-leader or even a higher qualified clinician such as ALS paramedic or doctor. A subset of staff also expressed the importance of these discussions being recorded.

BOX 1 Key themes in transport decision-making and patient management.

1. Shifting decision-making responsibility to specialised team, police, senior manager or clinician or family
2. Sedation or physical restraint
3. Risk assessment steps
4. Family involvement
5. Involuntary care provision or omission

Family, involvement was utilised in various means. This included using the family to convince the patient to be transported by ambulance voluntarily and in certain cases requested the family transport the patient by force privately to hospital. In the vignettes involving minors with suicide attempts, the respondents reported that they would allow family to make the ultimate decision about transport. Some participants described the family as being a hindrance rather than a help to the prehospital care providers’ management strategies: ‘they get in the way’ and ‘the family are sometimes more difficult than the patient’.

The provision of involuntary care to patients who pose a potential danger to themselves was not unanimously expressed by respondents. In the vignettes, less than half of respondents stated that they would transport a patient to hospital against their will. ‘I can’t force the patient … I have to leave patient at home. When the patient refuses transport there is not much I can do’ ‘We can’t force anyone because it is a form of kidnap’. The importance of the patient signing for their refusal of care was commonly reported and many expressed the belief that this documentation absolved them from legal responsibility.

### Personal experiences and attitudes around the management of suicide attempt patients

A portion of respondents expressed negative attitudes towards patients, citing varying reasons. A common theme identified was that caring for these patients was a ‘waste of time’, with prolonged time spent ‘talking in circles’ for which they felt they did not have the patience or would be penalised in terms of performance. There was mention that the time ‘wasted’ with these patients could be better spent. ‘It feels like you are able to help more people with your time’. Perceived self-pity on the patient’s behalf was seen as an irritation by some respondents: ‘clearly looking for attention, trying to spite somebody and just wasting people’s time if they weren’t going to do any harm to themselves … pretending’. Emotions expressed included feeling drained from these encounters, scared, threatened, finding it difficult to not be judgemental and an inability to empathise. Some respondents expressed that the patient has ‘chosen to die’ and should therefore be left to their own devices.

Numerous prehospital providers report that they feel uneasy about the uncertainty of the outcome of their suicidal patients who were not transported to hospital. Some reported to actively avoid obtaining follow-up on these patients for fear of the consequences. At least five respondents reported a personal account of a suicidal patient’s death after being given permission to sign a refusal of care or transport document and being left on scene. Additionally, four respondents reported having not transported patients to hospital, who subsequently developed severe complications resulting from the suicide attempt or a subsequent attempt at suicide. In addition, 15 respondents mentioned that they personally know a colleague who reported a death of a suicidal patient after not transporting the patient to hospital. Staff reported a lack of support after these events with a lack of counselling or debriefing opportunities. One reported a colleague resigning because of difficulty in coping with a patient death.

More than half of participants identified threats to their safety during the care of suicidal patients as a concern. Three reported having been injured by suicidal patients in the course of emergency assessment and treatment. Mechanisms of injury included bite wounds, facial scratching and blunt assault. Reports of self-defence methods utilised by staff included the use of a Taser as well as placing the patient in a ‘head-lock’ until assistance arrived.

Participants described concern regarding the risk of a patient committing suicide while in their care in the ambulance. Two respondents described past experiences of patients stabbing themselves and one respondent reported a patient jumping from a moving ambulance.

### Training of prehospital care providers in the context of the *Mental Health Care Act*

Of the respondents, 80% (*n* = 78) reported no training in the management of psychiatric patients, while only 7% (*n* = 7) had specific training in the *MHCA* of 2002. A chi-square test reveals no association between qualification and training in psychiatric management (*p* = 0.062) or the *MHCA* (*p* = 0.41). Participants expressed a desire for further education or training in the management of psychiatric (and specifically suicidal) patients in the form of Continuous Medical Education (CME) sessions, internal service updates or training as part of original qualifications.

## Discussion

‘More focus could be put on the actual patient instead of RHT [*Refusal of Hospital Treatment*] documentation. More thought could be put towards actually helping the patient in need instead of only worrying about paperwork.’ (Participant 109, male, private sector)

When considering the way prehospital providers approach the management of suicidal patients, numerous themes emerged. Patients who presented with minimal traditional risk factors for completed suicide were managed more conservatively and were less likely to be transported to hospital against their will. This is of major concern considering that patients with minimal risk factors are still inherently at risk of potential self-harm or suicide. Eventual death by suicide by these patients is demonstrated in the literature,^8^ and specific accounts of eventual suicide in patients left on scene have been reported by the respondents sampled in this study.

Respondents erroneously mentioned that patients cannot be transported to hospital without consent under any circumstances. Respondents did not have the confidence to make decisions related to the further management by delegating the transport decisions to the family, specialists or senior role-players. We have identified a significant need for training of South African prehospital providers with regard to the *MHCA* as well as on-scene evaluation of the suicidal patient. In many countries, trained prehospital providers may initiate involuntary mental health holds.^12^

Personal safety was an important theme identified. In a recent unpublished study on prehospital personnel, 56.0% of the 158 participants reported that they had been assaulted while on duty.^15^ Similar results are described in the international literature that report an incidence of violence between 61.0% and 87.5%.^16^ To protect themselves from potentially dangerous patients, providers often involve the police. Providers expressed concern that patients with negative previous experiences with police restraint often reacted poorly.

Of concern is the negative attitudes towards suicidal patients.^17^ Literature suggests that these often present as a lack of empathy on the part of the health care provider and can lead to accusing patients of attention-seeking behaviour.^18^ General lack of training, lack of decision-making support and fear of personal safety may contribute to these negative attitudes.

There was limited reported training across all qualifications with regard to *MHCA* and management of suicidal patients. Steps for improvement include: re-evaluating the current student curricula to better serve practical application and designing training programmes for already-graduated providers. Guidelines and policies for prehospital providers to assist in decision-making related to transport refusal in suicidal patients should be developed at a national level. Dedicated response teams with specific training may be a solution to manage these cases.

## Limitations

External validity and generalisability of the study were affected by the limited geographic nature, convenience sampling and the biases of self-selection. However, we feel that the depth of the personal experiences disclosed has significant value. The mental health of each of the participants was not assessed, and therefore the impact that this might have had on the specific transport decision-making cannot be extrapolated.

## Conclusion

This study shows a critical lack in the knowledge and training of prehospital providers within the Western Cape regarding the management of patients with a suicide attempt. Of further concern are the negative feelings expressed towards suicidal patients and a lack of commitment to transport patients to definitive care. The future development of, and research on, mental health care training programmes for emergency services and the adjustment of current curricula are critical. Specifically, this should focus on the transport of patients with emergency mental health care needs, methods of involuntary transport, psychiatric differential diagnoses and the legal framework within which this may be executed to protect the rights of the patient.
